# Investigation of Sub-100 nm Gold Nanoparticles for Laser-Induced Thermotherapy of Cancer

**DOI:** 10.3390/nano3010086

**Published:** 2013-01-31

**Authors:** Jennifer P. Leung, Sherry Wu, Keng C. Chou, Ruth Signorell

**Affiliations:** 1 Department of Chemistry, University of British Columbia, 2036 Main Mall, Vancouver, BC V6T 1Z1, Canada; E-Mails: leungj1@mail.ubc.ca (J.P.L.); sherryshwu@gmail.com (S.W.); kcchou@chem.ubc.ca (K.C.C.); 2 ETH Zurich, Laboratory for Physical Chemistry, Wolfgang-Pauli-Strasse 10, CH-8093, Zürich, Switzerland

**Keywords:** gold nanorods, gold core-corona nanoparticles, hollow gold nanoshells, light scattering microscopy, prostate cancer, gene therapy

## Abstract

Specialized gold nanostructures are of interest for the development of alternative treatment methods in medicine. Photothermal therapy combined with gene therapy that supports hyperthermia is proposed as a novel multimodal treatment method for prostate cancer. In this work, photothermal therapy using small (<100 nm) gold nanoparticles and near-infrared (NIR) laser irradiation combined with gene therapy targeting heat shock protein (HSP) 27 was investigated. A series of nanoparticles: nanoshells, nanorods, core-corona nanoparticles and hollow nanoshells, were synthesized and examined to compare their properties and suitability as photothermal agents. *In vitro* cellular uptake studies of the nanoparticles into prostate cancer cell lines were performed using light scattering microscopy to provide three-dimensional (3D) imaging. Small gold nanoshells (40 nm) displayed the greatest cellular uptake of the nanoparticles studied and were used in photothermal studies. Photothermal treatment of the cancer cell lines with laser irradiation at 800 nm at 4 W on a spot size of 4 mm (FWHM) for 6 or 10 min resulted in an increase in temperature of ~12 °C and decrease in cell viability of up to 70%. However, *in vitro* studies combining photothermal therapy with gene therapy targeting HSP27 did not result in additional sensitization of the prostate cancer cells to hyperthermia.

## 1. Introduction

Nanotechnology is at the forefront of several advancements in medicine, as nanomaterials have the potential to improve existing or develop novel treatment methods for diseases, such as cancer. There are several treatment options for cancer, such as surgery, radiation, chemotherapy, cryotherapy and high intensity ultrasound [1]. However, most of these treatments are only partially effective, fail to avoid recurrences and/or are invasive [1,2,3]. Prostate cancer is one of the most common cancers among men [4,5] and is generally difficult to treat, as the prostate lies at a critical juncture between several major organs [1]. Available treatments often have a high risk of damaging the surrounding organs or have other adverse effects [2,3,6]. Photothermal therapy using gold nanoparticles is under development, as it can be used to treat cancer in a non-invasive or minimally invasive manner and potentially improve treatment outcomes [7,8,9].

Photothermal therapy is a treatment modality that uses light to destroy cancer cells by heat (hyperthermia). Light in the NIR is used, as tissue has the lowest absorption in this region [10,11]. Photothermal therapy operates in two basic modes: light only or light coupled to a photothermal agent. The use of a photothermal agent, such as gold nanoparticles, allows the generated heat to be localized and intensified, confining damage strictly to the area of interest. The effectiveness of the photothermal therapy depends on the properties of the nanoparticles, which determine the absorption of light and conversion of light to heat [12]. The optical properties of gold nanoparticles depend on their plasmon resonance, which can be tuned based on particle size, shape, architecture and environment [9,13,14]. Numerous particle types that absorb in the NIR have been developed for use in photothermal therapy [15], such as nanoshells [9], nanorods [16] and gold nanocages [17]. Large nanoshells (>100 nm) with a solid core are generally the most commonly studied in *in vitro* and *in vivo* cancer models [9,18]. The most prominent work is the commercialization of a 150 nm gold nanoshell (silica core of 120 nm and 15 nm gold shell) based therapy called AuroLase® developed by Nanospectra Biosciences Inc. (Houston, TX, USA) This therapy is currently in Stage I clinical trials testing intravenous delivery of the particles to ablate cancerous tumours in the head and neck [19]. This work proves the feasibility of a nanoparticle based photothermal therapy. However, although these large nanoshells have good heating behaviour [18], their size is not optimized for this purpose. Larger particles scatter a larger fraction of light and require more energy to heat the particle, which leads to less heat dissipation to the surroundings [20]. In general, smaller nanoparticles are more attractive, because absorption is maximized and a greater temperature rise is possible due to the smaller mass of gold. Nanoshells <100 nm in size have been studied far less, with only a few synthesis reports of nanoshells with diameters of 10 to 60 nm [21] and only one report of a photothermal application [22]. Depending on whether the particles are internalized into cancer cells by endocytosis or anchored to the outer cell membrane, particle size may be important. Chithrani and Chan determined that a particle size of 50 nm had the best cellular uptake, leading to the highest accumulation in the cell [23]. In this study, nonspecific endocytosis was the mechanism of cellular internalization. Therefore, the amount of particles accumulated in the cell will also determine the effectiveness of the therapy, as the generated heat is due to collective heating by the particles [12]. The prospect of improved uptake and more efficient light absorption make smaller particles more desirable for use in photothermal therapy. 

A limitation to the hyperthermic destruction of cancer is that cancer cells often have high thermotolerance [24]. Cells have many defensive mechanisms that protect the cell from environmental or physiological stresses. Heat shock proteins (HSPs) are part of the defence system and are essential to cell survival [25], as they facilitate the transport, folding and assembly of proteins. In particular, HSP27, a low molecular weight HSP, has increased expression in prostate cancer cells and is associated with thermotolerant and cytoprotective functions [24,26,27]. The level of HSP27 can also indicate prostate cancer progression. Work by Cornford *et al.* has suggested that the expression of HSP27 can be used as a diagnostic tool to predict the clinical outcome of patients with prostate cancer [28]. The cancer models used in this study were LNCaP and PC3 prostate cancer cell lines. These are commonly used in experimental work, as they can closely reflect disease conditions, as they can represent early and late stages of prostate cancer [29]. PC3 is an androgen-independent cell line, and LNCaP is androgen-dependent [30]. HSP27 is expressed in PC3 cells [24] and is weakly expressed in LNCaP [28]. Overexpression of HSP27 in LNCaP also causes the cells to become androgen-independent [31]. The presence of HSP is known to improve cell resistance to apoptosis and necrosis [25], which may result in a higher, more invasive temperature requirement to cause hyperthermia. Therefore, photothermal treatment efficiency may be improved if combined with strategic removal of HSPs by gene therapy. Removal of HSP can be accomplished by gene therapy using antisense oligonucleotides (ASO). ASO are single strands of nucleic acids that can be designed to be complementary to a specific sequence of RNA [32,33]. Introduction of ASO can result in the hybridization with the target RNA, blocking the synthesis of protein.

Multimodal or combination therapy represents a new approach in fighting disease. The benefits of integrating photothermal therapy with gene therapy are that the combination could supplement and/or support cancer cell death. Studies have also shown that HSP suppression increases sensitivity to various types of cancer treatments, such as chemotherapy [26,34,35] and radiation [36]. However, there are limited studies that examine the relationship between HSPs and hyperthermia. Work by Gabai *et al.* showed that the knockdown of HSP72 increased the sensitivity of prostate cancer cell lines to hyperthermia [34]. Similar results were found by Rossi *et al.* with their work targeting HSP70 in HeLa cells [37]. Although research has been done using gold nanoparticles as a delivery agent for many types of biomolecules, such as ASO [32,38], there have not been any investigations into the therapeutic effect of ASO and photothermal therapy together. Furthermore, no study has yet examined the effect of HSP27 removal on the cell defence system to heat and if its absence could result in increased heat sensitivity. 

In the present study, a novel therapeutic strategy for prostate cancer is proposed consisting of the combination of photothermal therapy with ASO gene therapy with the overall goal to develop a treatment formulation that may be directly injected into tumour volumes and subsequently treated with a laser. The benefit of this approach is that the formulation will be concentrated within the tumour, and since tumour vasculature is physiologically distinct from normal healthy tissue [18], the gold nanoparticles will be retained, which will minimize damage to surrounding healthy tissue. The individual effects of each therapy were investigated, and the total effects were then evaluated. Various types of small gold nanoparticles with a size <100 nm that absorb in the NIR were synthesized, characterized and studied to determine their effectiveness as a photothermal agent. Smaller particles have distinct advantages over larger particles commonly used in photothermal therapy. This was evaluated by comparison with commercially available particles (Auroshells®, large nanoshells, Houston, TX, USA). Gold nanoparticle uptake (nonspecific) into PC3 and LNCaP prostate cancer cell lines was determined by light scattering microscopy to confirm internalization and allow for a comparison of uptake between different particles. Photothermal therapy was performed *in vitro* by laser irradiation at 800 nm, and the results were evaluated by measuring the effect on cell viability. 

## 2. Results and Discussion

### 2.1. Nanoparticle Synthesis and Characterization

**Figure 1:**
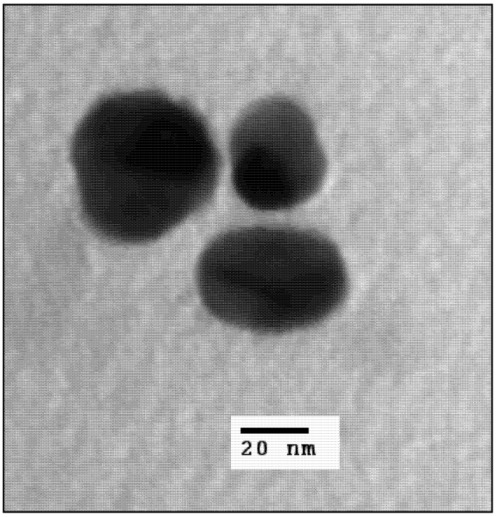
TEM image of 40 nm gold nanoshells.

**Figure 2:**
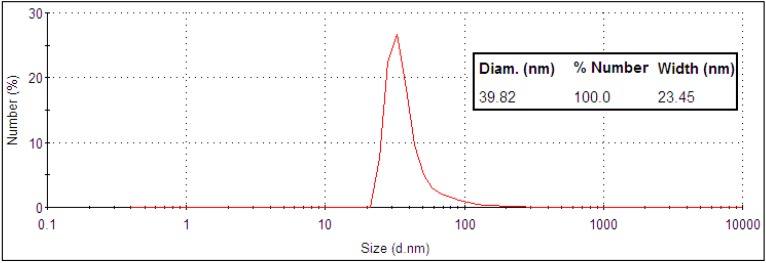
DLS size distribution data of 40 nm gold nanoshells.

A selection of gold nanoparticles with the appropriate size, stability and optical properties for use as a photothermal agent were investigated. Particles included were nanoshells, nanorods, core-corona nanoparticles and hollow nanoshells. All particles met the size criterion of <100 nm to maximize cellular uptake and absorption in the NIR to meet appropriate photothermal therapy conditions. Small gold nanoshells were synthesized with a 30 nm silica core and a shell thickness of 5 to 10 nm. The final product had a diameter of 40 ± 12 nm as, measured by TEM (Figure 1) and DLS (Figure 2).

**Figure 3:**
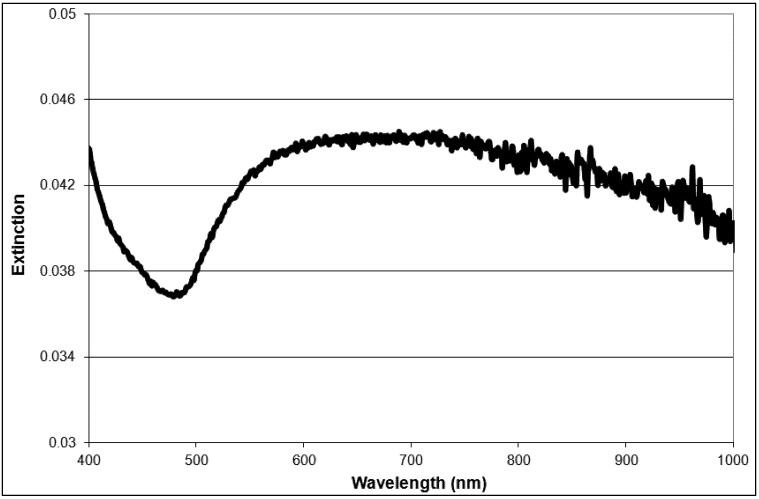
Extinction spectra of 40 nm gold nanoshells.

According to Mie theory, a 30 nm diameter silica core requires a core-to-shell ratio of 7 and a 2 nm shell thickness to generate a plasmon maximum at 790 nm [21]. Although the shell thickness generated in this work was at least 5 nm, the small nanoshells do exhibit a very broad, tail-end extinction in the NIR (Figure 3). This is consistent with the trends observed experimentally by Rasch *et al.* for nanoshells based on 28 nm and 38 nm silica cores [21]. The broadness and deviation from the expected optical behaviour was attributed to surface roughness, non-spherical particle shape, distribution in size and aggregation [21]. Rasch *et al.* suggests aggregation as a contributing factor to the shape of the extinction peak, as the coupling of plasmons leads to a lowering of the overall plasmon energy [39]. However, the size distribution of produced nanoshells shows a single peak for complete nanoshells (Figure 2). This result suggests fairly disperse particles in solution. The 30 nm silica cores were more spherical than the final nanoshell (Figure 1). Loss in uniform shape was attributed to the gold seeds. Variation in size distribution of the seeds and any clustering at the silica surface is expected to give a rough, uneven surface and a non-uniform shape [21]. Nanometer scale roughness has been shown to broaden the extinction of gold nanoparticles [18,40,41]. The extinction of small nanoshells in this work is expected to be influenced by all of the above factors. While the degree of influence for each factor is unknown, the net effect creates particles with a NIR absorption, which makes them appropriate for photothermal therapy. 

**Figure 4:**
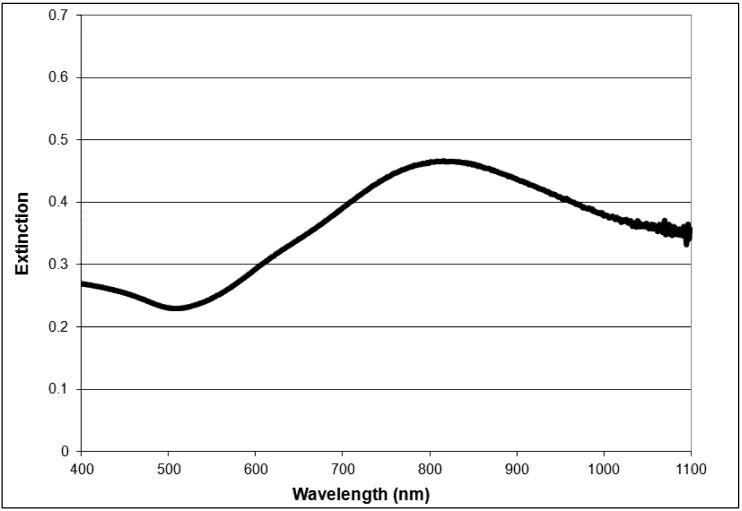
Extinction spectra of Auroshell® particles.

**Figure 5:**
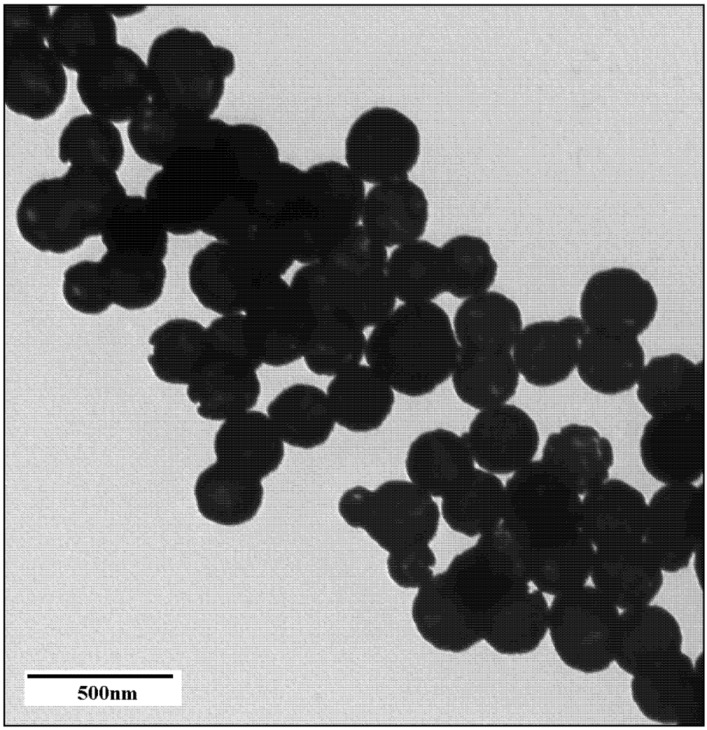
TEM image of Auroshell® particles.

**Figure 6:**
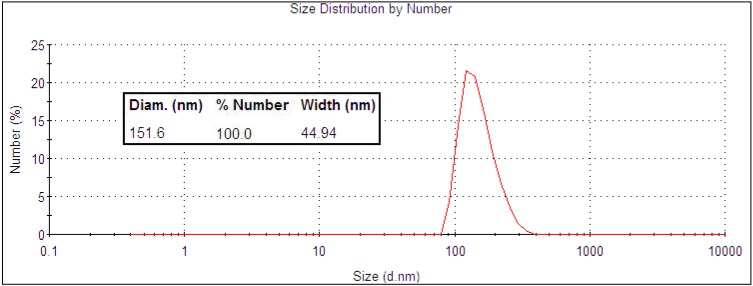
DLS size distribution data of Auroshell® particles.

The Auroshell® particles from Nanospectra Biosciences Inc. (large nanoshells; Houston, TX, USA) have a more distinct maximum in the NIR (Figure 4), as these particles are large enough for the extinction band to be tuned by the core-to-shell ratio. The extinction band is still quite broad, which is attributed to the same factors that affect the absorbance of the smaller 40 nm gold nanoshells. The size was measured using TEM (Figure 5) and DLS (Figure 6), and the values (152 ± 23 nm) were close to the reported size given by the manufacturer (silica core 120 ± 12 nm with a 15 nm shell thickness). 

Similarly, as the 40 nm gold nanoshells, all other particles investigated—nanorods (length = 73 ± 7 nm, width = 21 ± 2 nm), core-corona nanoparticles (78 ± 11 nm) and hollow nanoshells (85 ± 17 nm)—also demonstrated broad extinction spectra in the NIR and overall sizes below 100 nm (see the Supporting Information for further details). Quite a few approaches to generating gold nanoparticles exist, with wet chemical methods often being the preferred choice because of simplicity. Although the mechanisms governing particle growth for many gold nanoparticles are not well understood [42], most synthetic techniques, for common particle types, are well developed and easily controlled. This allows for a vast selection of particles and many methods to generate them. Generally, particles are selected and evaluated based on size, shape, presence of impurities/toxic reagents, optical and thermal stability and scalability for practical applications (see the Supporting Information for discussion). Of the selection of gold nanoparticles studied in the present contribution, small gold nanoshells (40 nm) exhibited the best overall properties for photothermal therapy of prostate cancer cells (see also following sections).

### 2.2. Light Scattering Microscopy and Cellular Uptake

Gold nanoparticles may be anchored to the outer membrane of cells or internalized, depending on the functionalization of the gold surface. In this work, uptake is referred to as internalization of the nanoparticles by the cells, and particles are taken up non-specifically, as they are not functionalized to target receptors on the cell membrane. All eukaryotic cells have a membrane system that allows the uptake of molecules and particles from extracellular fluid [43]. If the particles are internalized, rather than specifically attached to the surface, the chances of cell necrosis after photothermal treatment may be higher [44], as there is collective heating of the particles [12] within the cell. For photothermal therapy, the amount of gold nanoparticles taken up by the cells will determine the effectiveness of the treatment. Therefore, it is important to measure accumulation. The light scattering properties of gold nanoparticles can be used to image cancer cells and detect the particle uptake *in vitro*. Light scattering images were obtained in order to gage particle uptake as cells exhibit minimal scattering. Light scattering is not a quantitative measure of internalization, as the scattering efficiency changes with particle properties and it is difficult to quantify the scattering intensity. However, it still can provide a simple confirmation of cellular uptake and provide a qualitative comparison between different particle types.

**Figure 7:**
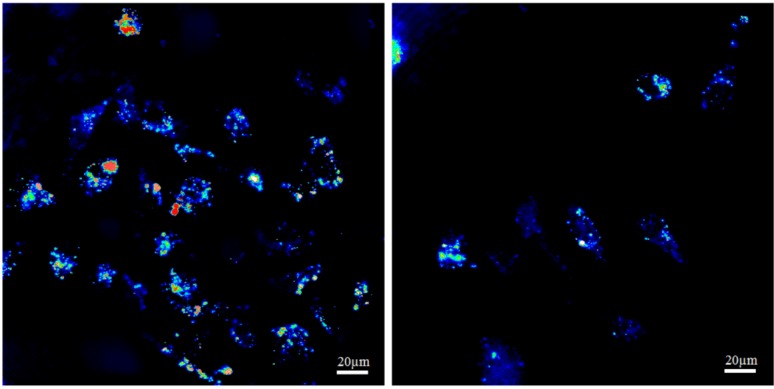
Cellular uptake comparison for 40 nm gold nanoshells (**left**) and Auroshell® particles (**right**) in PC3 cells.

Figure 7 shows representative scattering images of cells incubated with 40 nm nanoshells and Auroshells® (150 nm nanoshells) in PC3 cells. The particle incubation concentration determined by ICP-MS was approximately 4 × 10^12^ particles/mL for both particle types. Small nanoshells had the most consistent uptake, with accumulation across a greater portion of the cell (Figure 7 left). Although the larger Auroshell® particles are expected to scatter a greater fraction of light, the cells have lower scattering intensity (Figure 7 right) as compared to cells incubated with 40 nm nanoshells. This suggests that the smaller nanoshells do have a better cellular uptake. Several studies have found that smaller particles do show a higher accumulation in cells [23,44,45], which can support this claim. 

**Figure 8:**
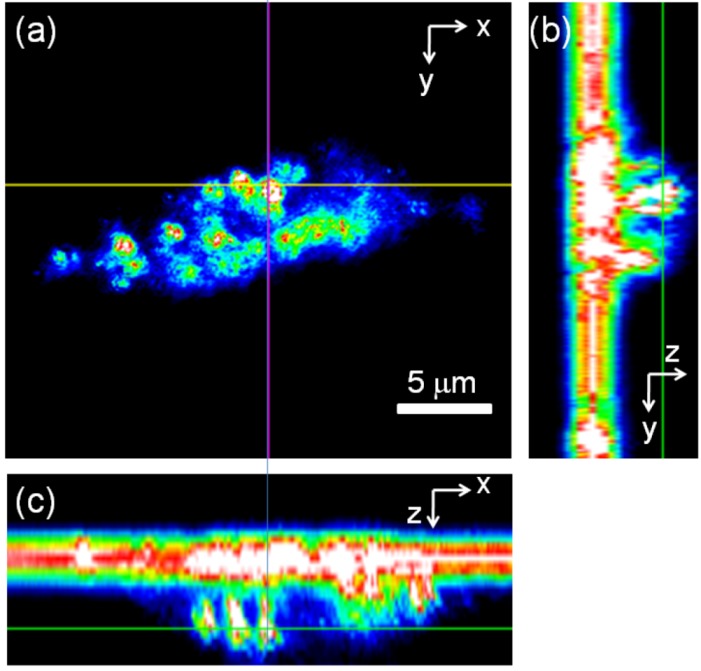
2D light scattering images of a single PC3 cell incubated with 40 nm gold nanoshells. (**a**) *x*,*y*-cross section; (**b**) *y*,*z*-cross section; (**c**) *x*,*z*-cross section. The thin lines mark the positions where the cross sections have been taken. The bright straight lines in (**b**) and (**c**) are reflections from the cover slide. The cell is visible in (**b**) to the right of and in (**c**) below the cover slide.

The three 2D images in Figure 8 are representative for the distribution of gold nanoshells within a single cell. The top left shows a *x*,*y*-cross section at a specific *z* position, while the right and bottom panels show a *y*,*z*-cross section at a specific *x* position and a *x*,*z*-cross section at a specific *y* position, respectively. The two glowing bands visible in the right and bottom panels are due to the reflection of the cover slip and the microscope slide and are thus not features of the cell. The cell is visible in between, attached to the cover slip. The images in Figure 8 clearly demonstrate that the particles are in the cell interior (*i.e.*, cytoplasm) and are not attached to the cell membrane. Further examination of various single cells at several different *x*, *y* and *z* positions proved that the particles are indeed internalized (Figure 8 shows only one representative example).

We found that the 40 nm nanoshells had the best internal particle distribution compared with other particles, including Auroshells® (Figure 7). Similar to the Auroshells®, the cellular uptake of nanorods, core-corona nanoparticles and hollow nanoshells were found to have far less internalization than the 40 nm nanoshells (data not shown). This result may be associated to size [23,44,45], shape [23,44] and surface properties [16,44,46]. Nanorods stabilized with a coating of CTAB were toxic to PC3 and LNCaP cells (see the Supporting Information for further details). Replacement of CTAB with a PEG coating resulted in minimal to no uptake, as PEG coated particles result in less interaction with the cell membrane, leading to a lower uptake [45,47].

### 2.3. ASO Gene Therapy

**Figure 9:**
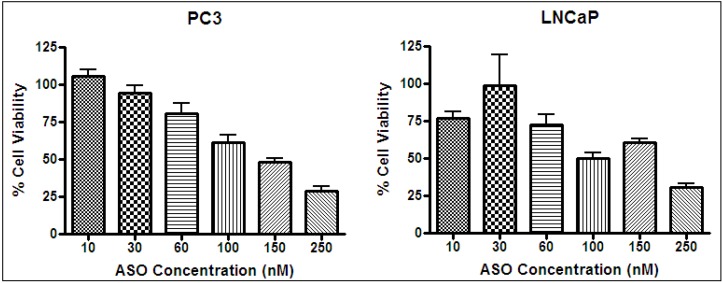
Antisense oligonucleotides (ASO) concentration test on PC3 (**left**) and LNCaP (**right**) at 37 °C. Cell viability by MTS assay.

**Figure 10:**
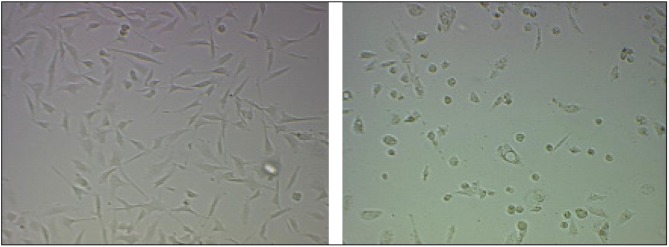
Microscope images of untreated (**left**) and transfected (**right**) PC3 cells. Treatment effect of 100 nM concentration of ASO.

Cells typically respond to lethal treatments in two ways: arrest in the cell cycle or the initiation of protective responses. When cancer cells are exposed to a heat shock, the latter type dominates, as there is a vast selection of pro-survival mechanisms [48]. Many of these mechanisms are mediated by HSP27 [26]. As a result of high heat resistance, unassisted thermal treatment of cancer must be done at high temperatures in order to be effective. Therefore, increasing the sensitivity of cancer cells to heat by gene therapy is an attractive option. The knockdown of HSP27 was done by an ASO (OGX-427) specific to HSP RNA. An ASO concentration test was performed, and it was determined that ASO had a concentration-dependent effect on cell viability (Figure 9). Based on cell viability results, the transfection of ASO using Lipofectamine on PC3 and LNCaP was effective, as the trend was consistent with work by Kamada *et al*. [26]. HSP27 ASO reduced the level of HSP27 and the corresponding cell viability in a dose-dependent manner. At a dosage of 50 nm, >95% of measured HSP27 was inhibited, as determined by Western blotting [26]. Knockdown of HSP27 did result in a noticeable change in cell condition (Figure 10), which can be explained by the work of Rocchi *et al*. [27]. They decreased HSP27 expression to undetectable levels in LNCaP and PC3 cells, resulting in a 2.4- to 4-fold increase in apoptotic cell death and 40%–76% inhibition of cell growth. 

### 2.4. *In Vitro* Photothermal Therapy and ASO Gene Therapy

Photothermal treatment, using gold nanoparticles and laser irradiation at 800 nm, was evaluated by measuring the changes in cell viability of PC3 and LNCaP cells using the MTT assay. The conditions for *in vitro* model experiments are selected to mimic therapeutic conditions for treatment in humans and must assess any limitations that may arise. For photothermal treatment, the absorption and scattering of light by tissue represents a treatment restriction that must be considered [10]. Major interferences in biological tissue are haemoglobin (Hb) and water (H_2_O), as they absorb visible and infrared light [11]. In the NIR, both species have the lowest absorption and scattering is minimized. *In vitro* studies can determine the basic requirements needed to destroy cancer cells. The photoinduced cell death of cancer using gold nanoparticles is dependent on the hyperthermic conditions generated. The conditions are determined by the amount of nanoparticles internalized or absorbed to the surface of cells [49,50], laser intensity [7,51] and total laser exposure time. Cheng *et al.* performed dosage studies on three cancer cell lines and found that there are minimum effective dosages that are dependent on the cancer cell line [50]. Laser irradiation studies of hybrid gold nanoparticles by Kirui *et al.* demonstrated that 53% of colorectal cells were destroyed upon a 6 min treatment with a laser intensity of 5.1 W/cm^2^ (4 mm spot size) and 99% for 31.5 W/cm^2^ [7]. The heating time is important, and the effect on cell viability is more noticeable for longer times [52]. However, in practice, it is not feasible to have a laser treatment time longer than a few minutes. Treatment times should typically be below 10 min [9,50].

**Figure 11:**
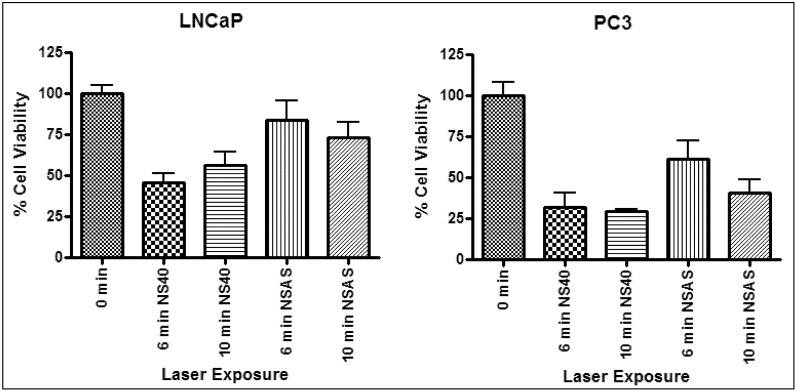
Representative photothermal experiment on LNCaP (**left**) and PC3 (**right**) incubated with 40 nm gold nanoshells (NS40) and Auroshell® particles (NSAS). Irradiated with a 4 W laser with a 42 °C background temperature.

The treatment results of the 40 nm gold nanoshells (NS40) were compared against the Auroshells® (NSAS). Photothermal treatment was done using comparable particle concentrations for incubation. The cell viability results for treatment with NS40 and NSAS are shown in Figure 11 for a 4 W laser exposure. A summary of the results is given in Table 1.

**Table 1:** Summary of the *decrease* in cell viability and changes in temperature for photothermal experiments with 40 nm gold nanoshells and Auroshell® particles in Figure 11.

Laser irradiation (min)	Sample	LNCaP			PC3		
		Δ Temp (°C)	Δ Cell viability (%)	*p* value	Δ Temp (°C)	Δ Cell viability (%)	*p* value
6	NS40	12 ± 1	54	<0.05	12 ± 1	68	<0.05
	NSAS	8 ± 1	27	<0.05	9 ± 1	39	>0.05
10	NS40	11 ± 3	44	<0.05	12 ± 1	71	<0.05
	NSAS	9 ± 1	35	<0.05	9 ± 2	60	<0.05

The gold nanoparticles did not exhibit any toxicity at the incubation concentrations used (data not shown). Therefore, the 40 nm gold nanoshells and Auroshells® can be considered to be non-toxic. Cells, devoid of gold, were not affected by the laser under the same treatment conditions. A maximum temperature rise of 5 °C was obtained for up to 10 min of exposure in the absence of gold particles. Laser irradiation resulted in an increase of up to 12 °C for samples incubated with 40 nm gold nanoshells and up to 9 °C with Auroshells®, for both PC3 and LNCaP. The decrease in cell viability in Table 1 is its change relative to the non-irradiated control devoid of gold (100%). The greatest cell viability decrease for LNCaP and PC3 was 54 and 71% (*p* < 0.05), respectively. This proves that an adequate amount of gold nanoparticles were internalized by the cells to generate a collective heating effect localized within cancer cells. Photothermal therapy with the 40 nm gold nanoshells resulted in a larger effect on cell viability and exhibited a greater change in temperature for both cell lines for all treatment times, suggesting a higher therapeutic efficiency compared to Auroshells®. This may be a result of the higher photothermal efficiency, greater cellular uptake (see Figure 7) or both. Treatment performed on PC3 did demonstrate a greater effect on cell viability as compared to LNCaP. This suggests LNCaP may have more effective survival mechanisms or might be a consequence of a greater gold nanoparticle uptake of PC3. The photothermal efficiency of pure concentrated solutions of 40 nm gold nanoshells and Auroshells® were calculated based on the ICP-MS concentration results (2.6 × 10^−^^4^ and 9.2 × 10^−^^5^ molAu/mL) [44]. Although the particle concentrations are not exactly equivalent, the measured absorbances of the nanoparticle solutions were close in value. At an irradiation power of 4 W for 1 min and temperature increases of 59 and 29 °C, the efficiencies were 21% and 10%, respectively. Therefore, the thermal conversion is almost double for the smaller nanoshells. The higher efficiency for 40 nm gold nanoshells is consistent with the fact that smaller particles generate and dissipate more heat [20].

**Figure 12:**
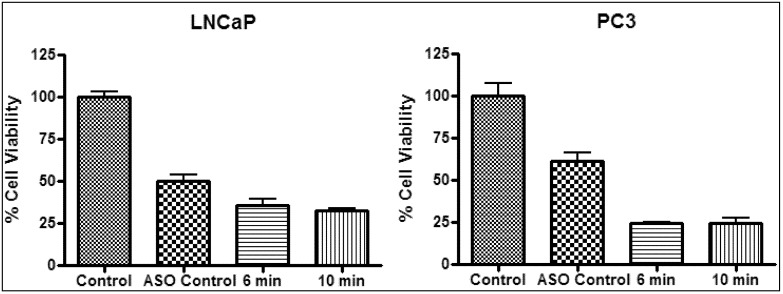
Representative photothermal experiment on LNCaP (**left**) and PC3 (**right**) treated with 100 nM ASO and 40 nm gold nanoshells. Irradiated with a 4 W laser with a 42 °C background temperature.

Photothermal treatment was combined with ASO gene therapy to study the net effect of the combined treatments (Figure 12). In the following, a “synergistic effect” means that the presence of ASO leads to a significant increase in heat sensitization for the combined treatment. Gene therapy with HSP27 ASO was found to reduce cell viability (Figure 9) by inhibiting cell proliferation and promoting apoptosis [27]. Treatment with 100nM HSP27 ASO resulted in an initial decrease in cell viability of ~50% and ~39% (*p* < 0.05) for LNCaP and PC3 (Table 2), respectively. Treatment of ASO transfected samples, in the absence of gold nanoparticles, with the laser for 6 and 10 min had no additional effect on cell viability (data not shown). Treatment with ASO and 40 nm gold nanoshells followed by laser irradiation for 6 min resulted in a total of ~64% decrease in viability for LNCaP and a total of 76% decrease for PC3 (Table 2). Laser radiation for 10 minutes resulted in a total of ~67% decrease in viability for LNCaP and a total of 76% decrease for PC3 (Table 2), respectively. When the NS40 results of Table 1 and Table 2 are compared, there is not a significant difference between NS40 and (ASO + NS40) treatments. For both cell lines, the effect of (ASO + NS40) is larger than the effect of ASO alone (in particular for the PC3 cells), and it is also larger than the effect of heat (NS40) alone, albeit not significantly. From our results, it is thus not clear whether or not the combination of ASO with photothermal therapy results in a synergistic effect by affecting the heat sensitivity of PC3 and LNCaP. However, the combined treatment at temperatures of ~53 °C resulted in the destruction of up to 76% of the cell population.

**Table 2:** Summary of the decrease in cell viability and changes in temperature for photothermal experiments with 40 nm gold nanoshells and 100 nm, as in Figure 12.

Sample	Laser irradiation (min)	LNCaP			PC3		
		Δ Temp (°C)	Δ Cell viability (%)	*p* value	Δ Temp (°C)	Δ Cell viability (%)	*p* value
ASO	-	-	50	<0.05	-	39	<0.05
ASO + NS40	6	11 ± 1	64	<0.05	9 ± 1	76	<0.05
ASO + NS40	10	11 ± 1	67	<0.05	10 ± 1	76	<0.05

## 3. Experimental Section

### 3.1. Materials

For all materials used, see Supplementary Information. 

### 3.2. Preparation of Gold Nanoparticles (<100 nm)

Gold nanoshells, nanorods, core-corona nanoparticles and hollow nanoshells were prepared by standard methods or modified procedures (see the Supporting Information for further details). Gold nanoshells (40 nm) were prepared using 30 nm aminated silica nanoparticles synthesized by a modified Stöber method [53,54]. The cores were seeded with small gold particles (<3 nm) [55,56], and the gold shell was produced by further reduction of gold [57]. Gold nanorods were prepared by the seed-mediated growth method by Nikoobakht and El-Sayed [58]. Gold core-corona nanoparticles were prepared using a modified procedure by Preston and Signorell [40]. The procedure was shortened by growing the corona directly on a silica core. Hollow gold nanoshells were synthesized by galvanic replacement of silver by gold. Growth of the gold shells was effected by a modified procedure by Au *et al*. to produce *in situ* polymer coated nanoparticles [59]. 

### 3.3. Particle Characterization

Particles were characterized by a Hitachi H7600 transmission electron microscope (TEM) operating at 80 kV and by dynamic light scattering (DLS) and zeta potential measurements using a Malvern Zetasizer Nano-ZS. TEM was used to determine the average particle size and examine particle shape. DLS were used to determine the average particle diameter for spherical particles. The zeta potential was used to measure surface charge to confirm particle coatings. Extinction spectra were measured with a Varian 50 Bio UV-visible spectrophotometer over the range from 400 to 1100 nm, using a 10 mm path-length quartz cuvette (Hellma, Müllheim, Germany). The gold nanoparticle concentration for nanoshells was determined by digesting the gold nanoparticles in aqua regia (1:3 volume ratio of HNO_3_:HCl, 2.5 mL) [60] and analyzing the total gold content by ICP-MS [44]. The ICP-MS analysis was performed by ALS Enviro, BC, Canada.

### 3.4. Cell Culture

Prostate cancer cell lines, LNCaP and PC3, were used to demonstrate cellular uptake and photothermal therapy under laser irradiation. Both are adherent cells that grow readily and are commonly used as experimental systems for *in vitro* and *in vivo* work of prostate cancer. The PC3 cells were cultured in Dulbecco’s modified Eagle medium (DMEM) and LNCaP cells in RPMI 1640 medium, supplemented with 10% fetal bovine serum (FBS) and 1% penicillin-streptomycin. For all studies, cells were grown to a confluence of 70% to 90%, at 37 °C and 5% CO_2_ in a humidified incubator.

### 3.5. Cellular Uptake by Light Scattering Microscopy

Cells were plated by adding approximately 1.5 × 10^5^ cells per well in a 6 well tissue culture plate containing 18 mm glass cover slips and grown to 70% to 90% confluence. Gold nanoparticles were added and incubated for 24 h at 37 °C under 5% CO_2_. Cells were incubated with nanoparticle solutions that had similar absorbance values. After incubation, the cells were fixed for 10 min in paraformaldehyde (2%, 1 mL). The cover slips coated with a layer of cells, were washed in PBS (1X, 1 mL) twice and mounted in 50% glycerol in 1X PBS on a microscope slide. Light scattering microscopy was used to image the gold particles using a previously developed set-up [61]. In brief, images were taken by using a modified Olympus FV300 laser-scanning microscope with a laser centered at 532 or 580 nm at a power typically less than 0.5 mW. The input laser beam was linearly polarized, and only the cross-polarized scattered light was collected using a 60× objective lens (NA = 1.2). The light intensity was detected by a photomultiplier tube (PMT) and digitized by an analog-to-digital converter. Two- (2D) and three-dimensional (3D) images of the cells were taken to observe uptake. 2D cross sections are shown as representative examples. 3D images were obtained by capturing a stack of 2D (*x* and *y*) images along the *z*-axis to determine if the particles were internalized by the cells (not shown). The lateral spatial resolution of the set-up was about 250 nm.

### 3.6. ASO Gene Therapy

The effect of ASO concentration on cell viability was performed by the MTS assay. PC3 and LNCaP cells were plated by adding 10,000 cells per well in a 96 well plate and incubated overnight (Day 1). The transfection (Day 2) was performed using Lipofectamine and different concentrations of ASO under serum-free conditions using Opti-MEM medium. Control experiments using pure Lipofectamine at incubation concentrations did not have an effect on cell viability, so the carrier system was considered to be non-toxic. Final ASO incubation concentrations were 10, 30, 60, 100, 150 and 250 nM. Final well volumes were 90 µL. After a 4 to 5 h incubation period, the medium was replaced with serum media and incubated for 15 h. MTS reagent (20 µL) was added to wells incubated for 3 h, and the optical density was measured directly at 450 nm (Day 3). The cell viability data was subject to statistical analysis using analysis of variance (ANOVA) with a *p* value of 0.05. ANOVA and a Bonferroni’s multiple comparison tests were performed using Graphpad Prism 4. When two or more samples were compared, if *p* < 0.05, the difference between the results was considered to be significant and if *p* > 0.05, the difference was not significant.

### 3.7. Photothermal Therapy

Cells were plated by adding 10,000 cells per well in a 96 well plate and incubated overnight (Day 1). ASO (100 nM) was transfected using Lipofectamine 2000 under serum-free conditions using Opti-MEM medium (Day 2). The total amount of Lipofectamine added was 5 μL per 250 μL of the total volume of ASO-Lipofectamine complex made, as per the manufacturer’s instructions. After a 4 h incubation period, the medium was replaced with serum media. Cells were incubated further for 15 h. Gold nanoparticles were added and incubated for 24 h (Day 3). Prior to the laser treatment (Day 4), all medium was removed, and fresh RPMI medium (100 to 200 µL) supplemented with FBS (10%) and HEPES (25 mM) was added. The cells, maintained at a set background temperature (42 °C) to mimic physiological conditions and to compensate for limitations in laser power, were irradiated with a continuous wave Ti:sapphire laser centered at 800 nm for 6 and 10 min [62]. The laser power and spot size were fixed to 4 W and 4 mm (FWHM). Cell viability after irradiation was assessed by the MTT assay. Various cell controls were prepared under similar conditions, without the addition of ASO, gold and application of the laser. The cell viability data was subject to statistical analysis, as previously described.

## 4. Conclusions

The present work reports on *in vitro* studies of the prostate cancer cell lines PC3 and LNCaP using photothermal therapy with gold nanoparticles and near infrared laser (NIR) irradiation in combination with gene therapy targeting heat shock protein (HSP)27 using antisense oligonucleotides (ASO). Various gold nanoparticles (gold nanoshells, gold nanorods, gold core-corona nanoparticles and hollow gold nanoshells) with strong absorptions in the near infrared and sizes below 100 nm were synthesized and evaluated according to their simplicity in synthesis, overall properties and cellular uptake into the prostate cancer cell lines. Larger nanoshells, 150 nm nanoshells (Auroshell®), were purchased from Nanospectra Biosciences Inc. (Houston, TX, USA) and used as a standard to compare cell internalization and photothermal treatment results *in vitro*. Overall, we find that small gold nanoshells (40 nm) have the advantage of the most straightforward synthesis, show the best cellular uptake and the highest change in cell viability upon photothermal treatment. The gold nanoshells were optically and thermally robust, as they were able to endure treatment times and temperatures. Smaller gold nanoshells exhibited better internal distribution throughout the cell and had a higher photothermal efficiency. This supports their greater impact on cell viability and higher change in temperature upon laser irradiation. Hyperthermic conditions were generated and easily controlled using these gold nanoshells to give a therapeutic dose of heat. Near infrared light and gold nanoshells are both individually not cytotoxic, as cell viability was unaffected by each treatment. However, when combined, irreversible destruction of cancer cells was achieved. Photothermal therapy with 40 nm gold nanoshells resulted in up to a ~70% decrease in cell viability at final temperatures of ~51 to 54 °C. 

It remains uncertain from our study whether the combination of photothermal therapy and ASO gene therapy results in a “synergistic” effect. The effect on cell viability of the combined therapy (decrease of up to ~76% at 53 °C) was found to be slightly larger than the effect of heat alone, albeit not significantly. However, treatment with ASO specific to HSP27 alone does exhibit a concentration-dependent effect on cell viability, which may be useful to overall treatment goals as a supplement to the treatment of cancer cells/tumours. Multimodal or combination therapy may be the best alternative to improve treatment conditions and outcomes for cancer. Further research is needed to explore the various options available to create an effective formulation that can be directly injected into tumours. Targeting a different HSP may be of interest or adding another complementary treatment, such as chemotherapy, as HSP27 knockdown is known to sensitize cancer cells to drug treatment [26,34,35]. There are several other aspects that need to be investigated before a complete treatment formulation can be developed, such as performing *in vivo* tests on animal models. Some preliminary *in vivo* work has been performed with gold nanoparticles alone, but protocols must also be put in place to analyze the treatment with ASO and to determine the efficacy of the combined treatment.

## Supplementary Files

Supplementary File 1
