# Supplementary material for: Investigation of Sub-100 nm Gold Nanoparticles for Laser-Induced Thermotherapy of Cancer
Source: Nanomaterials (Basel). 2013 Jan 31;3(1):86–106. doi: 10.3390/nano3010086 (PMC5304930; doi:10.3390/nano3010086)
Supplement: Supplementary File 1 [file nanomaterials-03-00086-s001.doc]

*Nanomaterials* **2013**, *3*, 86-106; doi:10.3390/nano3010086

**OPEN ACCESS**

***nanomaterials***

**ISSN 2079-4991**

www.mdpi.com/journal/nanomaterials

Article

Investigation of Sub-100 nm Gold Nanoparticles for
Laser-Induced Thermotherapy of Cancer

Jennifer P. Leung ^1^, Sherry Wu ^1^, Keng C. Chou ^1^ and Ruth Signorell ^1,2^,*

^1^ Department of Chemistry, University of British Columbia, 2036 Main Mall, Vancouver,
BC V6T 1Z1, Canada; E-Mails: leungj1@mail.ubc.ca (J.P.L.); sherryshwu@gmail.com (S.W.);
kcchou@chem.ubc.ca (K.C.C.)

^2^ ETH Zurich, Laboratory for Physical Chemistry, Wolfgang-Pauli-Strasse 10, CH-8093,
Zürich, Switzerland

***** Author to whom correspondence should be addressed; E-Mail: rsignorell@ethz.ch;
Tel.: +41-44-633-4621; Fax: +41-44-633-1316.

**Supplementary Information**

2. Experimental Methods

*2.1. Materials*

Tetrachloroauric(III) acid (HAuCl_4_⋅H_2_O, ≥99.9%), tetraethyl orthosilicate (TEOS, 98%), tetrakis(hydroxymethyl)phosphonium chloride (THPC, 80%), 3-aminopropyl-trimethoxysilane (APS, 97%), hexadecyltrimethylammonium bromide (CTAB, 99%), silver nitrate (AgNO_3_, 99%), L-ascorbic acid (C_6_H_8_O_6_), potassium carbonate (K_2_CO_3_, ≥99%), polyvinylpyrrolidone (PVP, MW = 55 kDa), trisodium citrate dihydrate (Na_3_C_6_H_5_O_7_·2H_2_O) and ammonium hydroxide (NH_4_OH, 28%–30%) were obtained from Sigma-Aldrich; sodium borohydride (NaBH_4_, 98%), sodium hydroxide (NaOH, 99.8%), hydrochloric acid (HCl, 36.5%–38%), nitric acid (HNO_3_, 65%–70%), formaldehyde (H_2_CO, 30%), paraformaldehyde (95%) and dimethylsulfoxide (DMSO, 95%) were obtained from Fisher Scientific; thiol-terminated polyethylene glycol (mPEG-SH, MW = 2000, 10,000 and 20,000 Da) were obtained from Laysan Bio Inc.; Auroshell® particles (2.894 × 10^9^ particles/mL) were obtained from Nanospectra Biosciences Inc.; Dulbecco's modified eagle medium (DMEM), RPMI 1640 medium, fetal bovine serum (FBS), penicillin-streptomycin, phosphate-buffered saline (PBS, pH 7.4), trypsin (0.25%), Opti-MEM I Medium, Lipofectamine 2000, RPMI Medium 1640 with 25 mM HEPES buffer and trypan blue stain (0.4%) were obtained from Invitrogen; CellTiter 96® Aqueous Cell Proliferation Assay (MTS) was obtained from Promega; thiazolyl blue tetrazolium bromide (MTT, 98%) was obtained from Alfa Aesar and used to prepare the MTT reagent (2.5 mg/mL in 1× PBS); PC3 and LNCaP (human prostate cancer cells) and HSP27 ASO OGX-427 (3.5 mM) were obtained from
Dr. Martin Gleave, Prostate Center, VGH, BC, Canada. All materials were used as received.

*2.2. Preparation of Small Gold Nanoshells (40 nm)*

Nanoshells were prepared by a set of previously developed procedures. Silica nanoparticles with a diameter of 30 nm were synthesized by a modified Stöber method [1,2]. To 50 mL of dry ethanol, ammonium hydroxide (28%–30%, 1.5 mL) was added. Under rapid stirring, TEOS (98%, 1.5 mL) was added and then stirred at 100 rpm for 8 hours. Amination was done by adding APS (97%, 24.5 µL) and stirring overnight. The cores were purified then seeded with small gold particles (<3 nm) [3,4]. The gold shell was completed by the reduction of gold [5]. Stock gold solution used to grow the shell was prepared at least 16 hours in advance by dissolving K_2_CO_3_ (100 mg) in water, adding HAuCl_4_ (1%,
6 mL) and adding water to a final volume of 100 mL. The coating was done by diluting different amounts of the gold solution in water to a final volume of 4 mL and adding the seeded silica cores (200 µL). Under mixing formaldehyde (30%, 10 µL) was added. The particles were centrifuged (14,000 rpm) for 30 min and redispersed in sterile water. The average diameter of the resulting particles was 40 ± 12 nm. This suggests a gold coating thickness of about 5 nm. UV-Vis absorption spectroscopy showed a broad peak that extended into the NIR. The concentration of the gold nanoshells produced was 2.2 × 10^11^ particles/mL.

2.3. Preparation of Gold Nanorods

*The nanorod synthesis was adapted from the seed-mediated growth method by Nikoobakht and
El-Sayed [6]. Gold seed particles were prepared by mixing an aqueous solution of HAuCl_4_ (1%,
100 µL), CTAB solution (0.20 M, 5 mL) and water (4.9 mL). To this ice cold NaBH_4_, (0.01 M,
0.6 mL) was added, and the solution was vigorously stirred for 2 min. Nanorod growth solution was prepared by mixing CTAB (0.20 M, 5 mL) with differing volumes (0.15, 0.20, 0.25 and 0.30 mL) of AgNO_3_ (4.0 mM). Under moderate stirring, water (4.8 mL) and HAuCl_4_ (1%, 200 µL) were added, followed by ascorbic acid (78.8 mM, 70 µL). The solution was maintained at a temperature of 25 to
30 °C. Nanorod growth was initiated by adding seed solution (12 µL) to the growth solution and stirring moderately for 5 min. Each solution gradually changed color after about 20 min. The
UV-Vis spectra confirmed the position of the extinction maxima, and TEM was used to determine the shape and size. Nanorods with an extinction maximum at ~800 nm had an aspect ratio of 3.5 to 4.0, with a length of 73 ± 7 nm and width 21 ± 2 nm. Particles were centrifuged (10,000 rpm) and redispersed in sterile water twice.* PEGylation of gold nanorods was done by a modified procedure of Liao and Hafner [7]. The twice centrifuged/redispersed nanorods (~9 × 10^10^ particles/mL, 0.5 mL) and PEG 10000 solution (5.0 mg/mL, 0.5 mL) were mixed with potassium carbonate (2 mM, 100 µL). The mixture was stirred overnight at room temperature and purified by dialysis. The UV-Vis absorption and zeta potential confirmed a successful coating.

*2.4. Preparation of Gold Core-Corona Nanoparticles*

A modified procedure used to generate core-shell-corona particles by Preston and Signorell [8] was performed. Instead of a gold core with polymer layers, a silica core was used to grow the corona. The procedure follows the same steps as outlined in the nanoshell synthesis, except for the shell growth stage. A more concentrated gold solution was used to grow the corona to promote the growth of
brush-like features. The seeded silica cores (200 µL) were added and the solution mixed before formaldehyde (30%, 20 µL) was added. Solutions were mixed for 1 to 2 days. The UV-Vis spectrum showed an extinction band in the NIR, and TEM confirmed the brush-like features and size of
78 ± 11 nm. Particles that exhibited the correct properties were centrifuged (14,000 rpm) for
30 min and redispersed in sterile water. Typical particle concentrations were 2.2 × 10^11^ particles/mL.

2.5. Preparation of Hollow Gold Nanoshells

Hollow gold nanoshells are synthesized by reducing gold by a galvanic replacement reaction onto a solid template. Spherical silver nanoparticles were used as the template and were prepared by the
well-known Turkevich method [9]. In a 100 mL round bottom flask, 50 mL water was heated in an oil bath. Silver nitrate (9 mg) was added to the flask, and the mixture was brought to a boil, before adding sodium citrate solution (1%, 1.0 mL). The solution was refluxed and stirred for at least 30 min. Generally, the color of the solution changed from colorless to yellow, then turbid gray, indicating the reaction was complete. The silver templates used had an average diameter of 60 nm and an absorbance maximum at 428 nm. Growth of the gold shells was done by a modified procedure by Au et al. to produce in situ polymer coated nanoparticles [10]. In a small round bottom flask, silver spheres
(2.5 mL) and PVP solution (0.1 g/mL in water, 7.5 mL) was added. The mixture was brought to a boil, and HAuCl_4_ (0.2 mM, 4.0 mL) was added rapidly. Upon addition of gold, the solution changes color to a blue/gray within seconds. The solution was refluxed for 5 to 10 min and purified by stepwise centrifugation. The UV-Vis spectra showed a broad extinction band with a maximum at 800 nm. The hollow gold nanoshells had an exterior diameter of approximately 85 ± 17 nm with an expected interior diameter around 60 nm. The particle concentration for a typical synthesis of hollow gold nanoshells was 3.4 × 10^8^ particles/mL.

3. Results

*3.1. Nanoparticle Synthesis and Characterization*

3.1.1. Gold Nanorods

Examination of TEM images for several syntheses returned at minimum 95% of rod-shaped particles, with the remaining 5% as cubes, spheres and other shapes (Figure S1). Stabilization without CTAB was achieved by coating with PEG. The optical spectra of CTAB-and PEG-coated gold nanorods are shown in Figure S2. The concentration of CTAB stabilized nanorods was calculated to be approximately 1.1 × 10^11^ particles/mL based on the extinction maxima (Figure S2). Coating with PEG 10,000 results in a red shift in the extinction maxima by ~40 nm; therefore, the spectra of nanorods immediately reveal whether they were coated. The zeta potential was also an indication of a complete coating. CTAB nanorods had a large positive potential, and PEG coated nanorods had a potential close to 0 mV. This proves CTAB was removed, because PEG creates a neutral surface by electrostatic shielding [11]. The synthesis of nanorods was simple and quick and produced particles with excellent optical properties. However, there is a great percentage of material wasted, as only ~15% of gold is predicted to form rods [12]. Also, a potential issue is photothermal reshaping of particles after heat treatment [13]. The work by Takahashi *et al.* revealed that after laser exposure, the absorption spectra of nanorods indicated some reshaping into spherical nanoparticles [14]. It is not well known whether CTAB concentrations needed to maintain particle stability are cytotoxic. If gold nanorods are produced on a large scale, complete detoxification may be difficult, as CTAB can be hard to remove. However, surfactant exchange and dialysis techniques show some promise [13].

**Figure S1.** TEM image of gold nanorods.


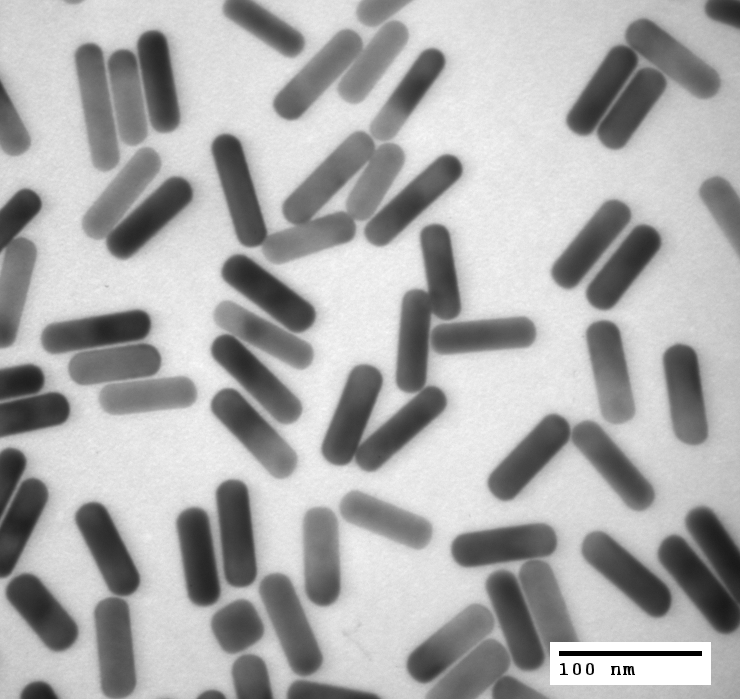


**Figure S2.** Extinction spectra of CTAB and PEG 10,000 coated gold nanorods.


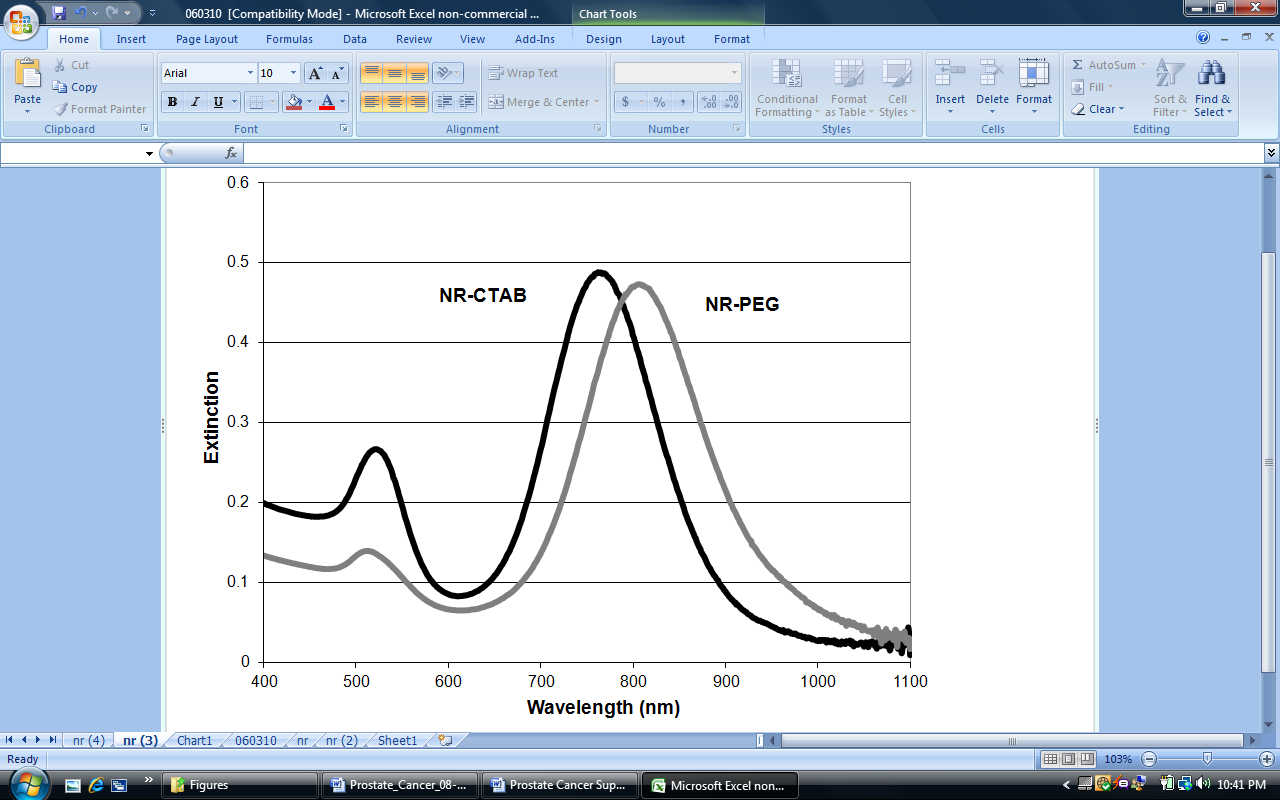


3.1.2. Gold Core-Corona Nanoparticles

The modified procedure to form core-corona nanoparticles was far less laborious than the synthesis route to generate the core-shell-corona particles [8]. The same brush-like features were obtained for the core-corona particles (Figure S3), and the optical properties (Figure S4) matched those of the
core-shell-corona particles, even without the core-corona plasmon contribution. Growth was not changed by the use of a different scaffold. Sizing by TEM shows the particles to be 78 ± 11 nm in size. Therefore, the corona thickness was about 25 nm, which is consistent with core-shell-corona particles generated by Preston and Signorell that absorb in the NIR [8]. These particles exhibit interesting and unique properties, with a strong absorption in the NIR (Figure S4). Although the original synthesis was simplified, the procedural method to generate the corona is not well-developed or optimized. Therefore, the route may still not be appropriate for a scale-up, unless further work is performed. The properties of these particles may have some similarities to gold nanoshells. However, the optical and thermal robustness of the particle is not known. The stability of the corona must be studied to see if this structural feature will suffer from collapse upon laser irradiation or heating. There is no documented data on the thermal efficiency of such a nanostructure and if cellular uptake is affected by a rough surface.

3.1.3. Hollow Gold Nanoshells

Generating hollow gold nanoshells by galvanic replacement of a solid template is a quick method to produce particles that absorb in the NIR. The method chosen here utilized simple reaction steps and minimized purification steps. Hollow gold nanoshells generated from these templates had an exterior diameter of approximately 85 ± 17 nm with an expected interior diameter around 60 nm. The shell thickness was not well defined for all particles; however, it can be estimated from the TEM to be
10–15 nm (Figure S5). It is evident that the nanoshells were hollow, as the centers look significantly lighter in the TEM image. This proves the silver template particle was removed. The optical properties of hollow nanoshells are very similar to those of solid core nanoshells, as both depend on the
core-to-shell ratio. However, the route to a hollow core has the potential to achieve thinner shells for smaller particle sizes [15]. The hollow gold nanoshells showed extinction bands in the NIR
(Figure S6). The synthesis was simple and generates gold nanoparticles with appropriate optical properties that can be used in photothermal applications. However, there are issues that need to be addressed before scale-up is considered. Silver and silver by-products are difficult to eliminate in the final nanoparticle product, and it has also been documented that complete removal of silver is not possible. Silver is still measured to be present, most likely incorporated in the hollow gold shell [16]. Work by Au *et al.* estimated that particles produced by the galvanic method contained up to 37%
silver [10]. Although silver does exhibit antimicrobial properties [16], it is unknown if this will be beneficial or harmful for *in vivo* use. Another issue that may affect the phototherapeutic use of these nanoparticles is that hollow structures have been proven not to be thermally robust. Short periods of laser irradiation collapses the nanoshells into smaller solid nanoparticles, resulting in an absorbance shift out of the NIR, limiting the nanoparticles active use in thermal therapy [16]. Whether these issues will produce problems downstream for medical applications will need to be further studied.

**Figure S3.** TEM image of gold core-corona nanoparticles.


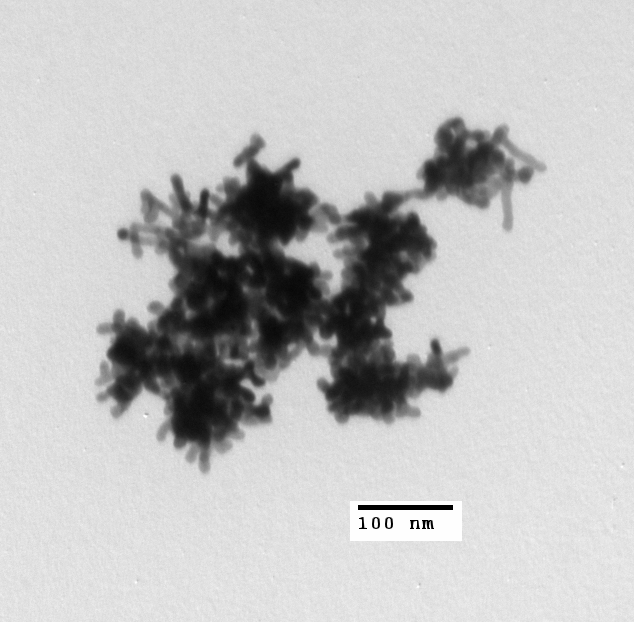


**Figure S4.** Extinction spectra of gold core-corona nanoparticles.


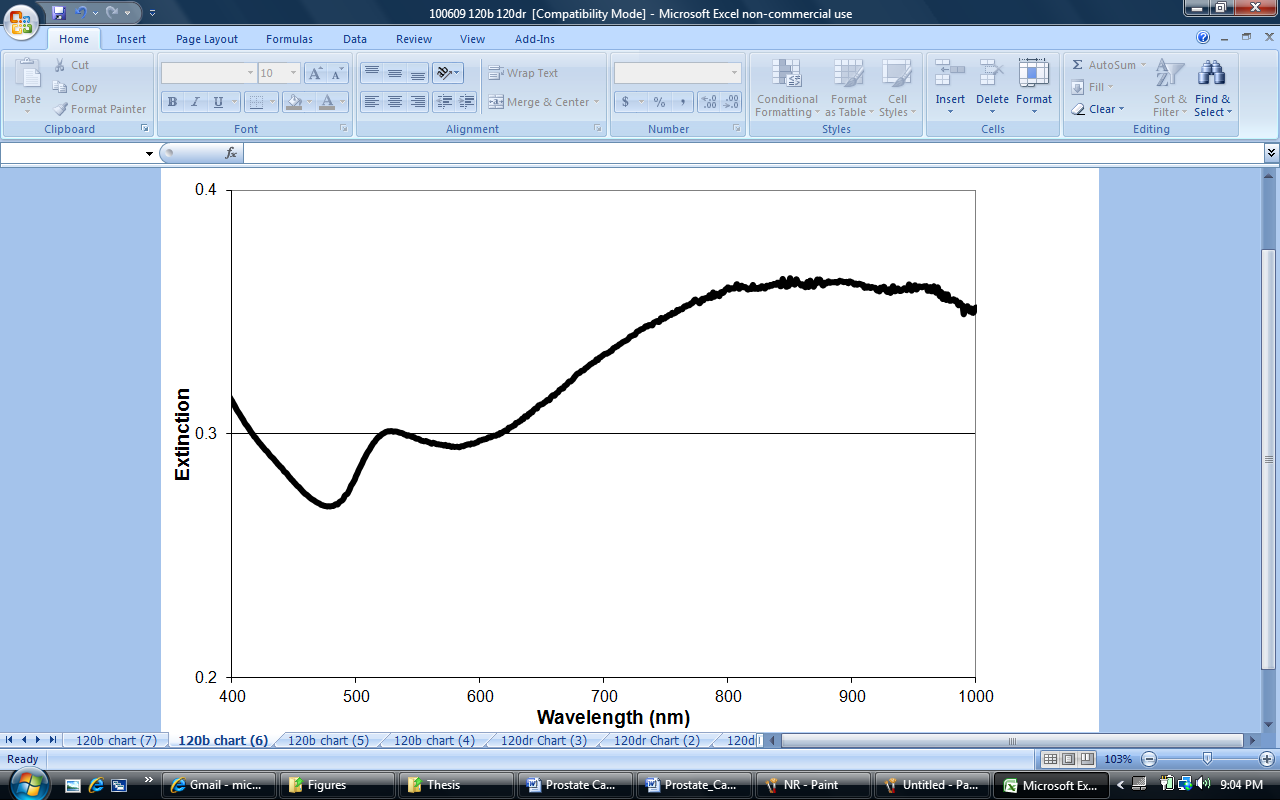


**Figure S5.** TEM image of hollow gold nanoshells.


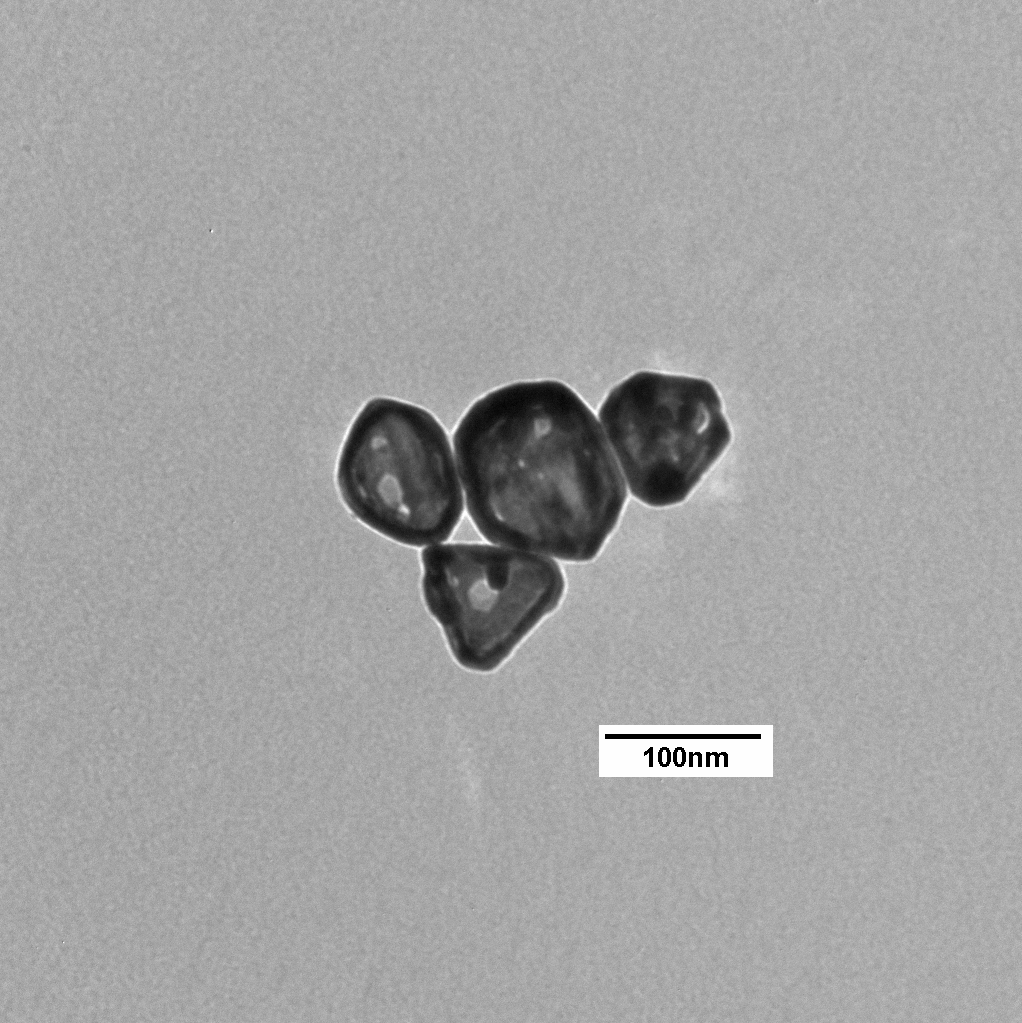


**Figure S6.** Extinction spectra of hollow gold nanoshells.


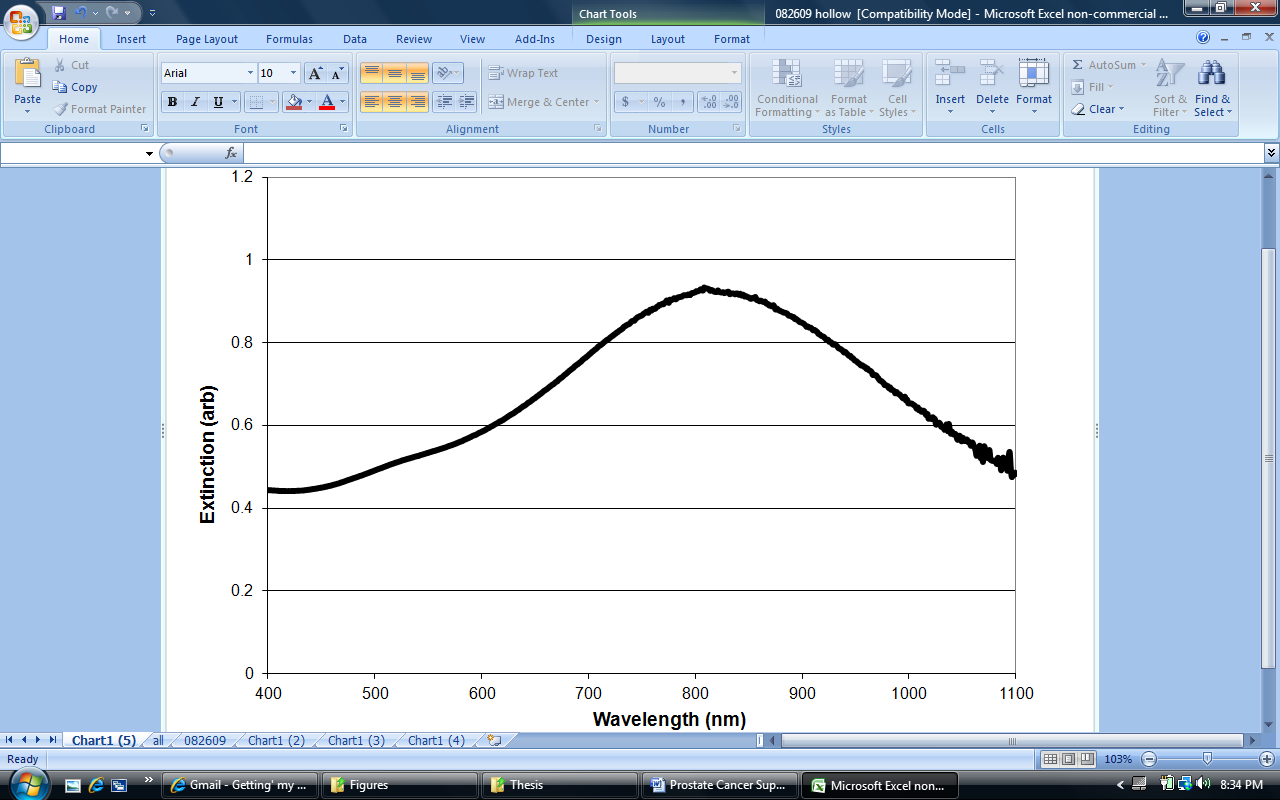


*3.2. Light Scattering Microscopy and Cellular Uptake*

Cells that were incubated with CTAB stabilized nanorods at an incubation concentration of
3 × 10^9^ particles/mL exhibited a toxic response. The cells were visibly unhealthy after 4 hours and by 24 h demonstrated cell death. Figure S7 illustrates the effect of CTAB on the cells. Healthy LNCaP cells were attached and spread across the surface (left) and unhealthy/dead cells were no longer attached and formed clusters (right). A similar response was observed in PC3 cells. Samples that were incubated with PEG-coated nanorods remained healthy for the entire duration of the incubation. Therefore, it can be assumed that CTAB does cause a cytotoxic response in PC3 and LNCaP cells at concentrations needed to keep the nanorods disperse in solution. Because of this result, only
PEG-stabilized nanorods were used for experiments.

**Figure S7.** Microscope images of LNCaP without (**left**) and with (**right**)
CTAB-coated gold nanorods after a 24 h incubation. Magnification: 100×.


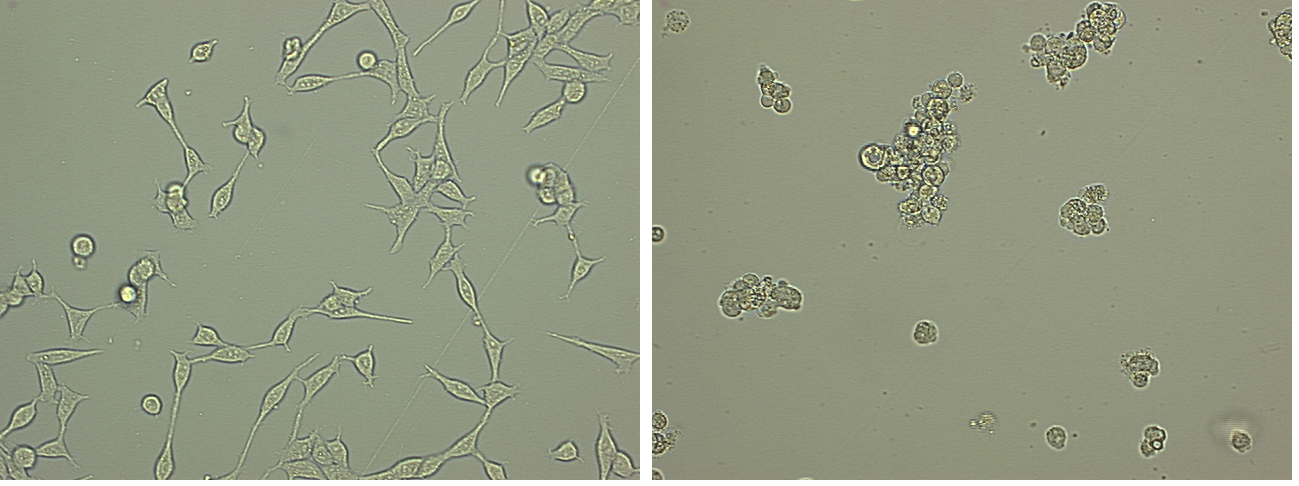


References

1. Stober, W.; Fink, A.; Bohn, E. Controlled growth of monodisperse silica spheres in micron size range. *J. Colloid Interface Sci.* **1968**, *26*, 62–69.
2. Sun, Y.Y.; Yan, F.; Yang, W.W.; Zhao, S.A.; Yang, W.S.; Sun, C.Q. Effect of silica nanoparticles with different sizes on the catalytic activity of glucose oxidase. *Anal. Bioanal. Chem.* **2007**, *387*, 1565–1572.
3. Duff, D.G.; Baiker, A.; Edwards, P.P. A new hydrosol of gold clusters 1. Formation and particle size variation. *Langmuir* **1993**, *9*, 2301–2309.
4. Oldenburg, S.J.; Averitt, R.D.; Westcott, S.L.; Halas, N.J. Nanoengineering of optical resonances. *Chem. Phys. Lett.* **1998**, *288*, 243–247.
5. Hirsch, L.R.; Halas, N.J.; West, J.L. Whole-Blood Immunoassay Facilitated by Gold
   Nanoshell-Conjugate Antibodies. In *Nanobiotechnology Protocols*; Rosenthal, S.J., Wright, D.W., Eds.; Humana Press: Totowa, NJ, USA, 2005; pp. 101–111.
6. Nikoobakht, B.; El-Sayed, M.A. Preparation and growth mechanism of gold nanorods (NRs) using seed-mediated growth method. *Chem. Mater.* **2003**, *15*, 1957–1962.
7. Liao, H.W.; Hafner, J.H. Gold nanorod bioconjugates. *Chem. Mater.* **2005**, *17*, 4636–4641.
8. Preston, T.C.; Signorell, R. Preparation and optical properties of metallodielectric
   core-shell-corona particles. *J. Phys. Chem. C* **2008**, *112*, 17844–17848.
9. Turkevich, J.; Stevenson, P.C.; Hillier, J. A study of the nucleation and growth processes in the synthesis of colloidal gold. *Discuss. Faraday Soc.* **1951**, *11*, 55–75.
10. Au, L.; Zheng, D.S.; Zhou, F.; Li, Z.Y.; Li, X.D.; Xia, Y.N. A quantitative study on the photothermal effect of immuno gold nanocages targeted to breast cancer cells. *ACS Nano* **2008**, *2*, 1645–1652.
11. Niidome, T.; Akiyama, Y.; Yamagata, M.; Kawano, T.; Mori, T.; Niidome, Y.; Katayama, Y. Poly(ethylene glycol)-modified gold nanorods as a photothermal nanodevice for hyperthermia.
    *J. Biomater. Sci. Polym. Ed.* **2009**, *20*, 1203–1215.
12. Orendorff, C.J.; Murphy, C.J. Quantitation of metal content in the silver-assisted growth of gold nanorods. *J. Phys. Chem. B* **2006**, *110*, 3990–3994.
13. Tong, L.; Wei, Q.S.; Wei, A.; Cheng, J.X. Gold nanorods as contrast agents for biological imaging: Optical properties, surface conjugation and photothermal effects. *Photochem. Photobiol.* **2009**, *85*, 21–32.
14. Takahashi, H.; Niidome, T.; Nariai, A.; Niidome, Y.; Yamada, S. Photothermal reshaping of gold nanorods prevents further cell death. *Nanotechnology* **2006**, *17*, 4431–4435.
15. Schwartzberg, A.M.; Olson, T.Y.; Talley, C.E.; Zhang, J.Z. Synthesis, characterization, and tunable optical properties of hollow gold nanospheres. *J. Phys. Chem. B* **2006**, *110*, 19935–19944.
16. Prevo, B.G.; Esakoff, S.A.; Mikhailovsky, A.; Zasadzinski, J.A. Scalable routes to gold nanoshells with tunable sizes and response to near-infrared pulsed-laser irradiation. *Small* **2008**, *4*, 1183–1195.

© 2013 by the authors; licensee MDPI, Basel, Switzerland. This article is an open access article distributed under the terms and conditions of the Creative Commons Attribution license (http://creativecommons.org/licenses/by/3.0/).
